# Long-term treatment with biosimilar epoetin-α (HX575) in hemodialysis patients with renal anemia: real-world effectiveness and safety in the MONITOR-CKD5 study 

**DOI:** 10.5414/CN109245

**Published:** 2017-11-23

**Authors:** Gérard London, Johannes Mann, David Goldsmith, Christian Combe, Frank Dellanna, Philippe Zaoui, Nadja Hoebel, Andriy Krendyukov, Karen MacDonald, Ivo Abraham

**Affiliations:** 1 Centre Hospitalier F.H. Manhés, Fleury-Mérogis, France,; 2 Friedrich Alexander Universität Erlangen-Nürnberg, Erlangen, Germany,; 3 Guy’s and St Thomas’ NHS Foundation Hospital, London, UK,; 4 Centre Hospitalier Universitaire de Bordeaux and Unité INSERM 1026, University of Bordeaux, Bordeaux, France,; 5 Dialysezentrum, Düsseldorf, Germany,; 6 Université de Grenoble, Grenoble, France,; 7 Hexal AG, Holzkirchen, Germany,; 8 Matrix45, Tucson, AZ, USA, and; 9 University of Arizona College of Pharmacy, Tucson, AZ, USA

**Keywords:** biosimilar, epoetin-α, erythropoiesis stimulating agents, renal anemia, HX575

## Abstract

Aims: To assess real-world effectiveness and safety of intravenous (IV) HX575, a biosimilar epoetin-α, in hemodialysis (HD) patients. Materials and methods: This prospective, observational, pharmacoepidemiological study of adult HD patients treated with IV HX575 for renal anemia for up to 24 months was conducted in 114 centers in 10 European countries. Of 2,086 enrolled subjects (safety sample), 2,023 had ≥ 1 follow-up visit (effectiveness sample). Results: Most (59.3%) patients were male, median age was 68 years. At enrollment, most (82.5%) had been treated with an erythropoiesis-stimulating agent, and 73.0% had adequate iron stores. At baseline, mean (± standard deviation) baseline hemoglobin (Hb) was 11.09 (± 1.14) g/dL and HX575 dose 106.5 (± 78.7) international units (IU)/kg/week; at month 24, Hb was 11.25 (± 1.19) g/dL and HX575 dose 113.0 (± 102.5) IU/kg/week. Variations in mean HX575 dose and Hb over the study were not statistically significant. As to safety, 140 patients (6.7%) experienced ≥ 1 adverse event; of these, 19 events (16 patients; 0.8%) were related to HX575 treatment, 148 (108 patients; 5.2%) were reported as serious, including 12 events in 11 patients (0.5%) stated to be related. No cases of anti-epoetin antibodies or pure red cell aplasia were reported. Conclusions: MONITOR-CKD5 confirmed the real-world effectiveness and safety profile of IV biosimilar HX575. HD patients treated for up to 24 months showed stable dosing patterns and Hb outcomes. The safety profile of HX575 is likewise comparable to reference epoetin-α.

Supplemental material is available for free at: http://www.clinnephrol.com Vol. 89 – January 2018

## Introduction 

Renal anemia is a major complication of chronic kidney disease (CKD), predominantly due to the failure of the kidneys to produce sufficient endogenous erythropoietin to stimulate the bone marrow to produce red blood cells (RBC) [1, 2]. Renal anemia is commonly corrected and subsequently maintained with erythropoiesis-stimulating agents (ESA). The most recent European Renal Best Practice [3] and Kidney Disease Improving Global Outcomes [4] guidelines recommend that ESA treatment be initiated at hemoglobin (Hb) levels below 10 g/dL but be withheld when Hb reaches 11.5 g/dL or higher. 

HX575 [5, 6, 7, 8, 9, 10], commercially available as Binocrit^®^ and Epoetin Alfa HEXAL^®^ (Hexal AG, Holzkirchen, Germany), was among the first biosimilars approved by the European Medicines Agency (EMA). The late-stage clinical development program for HX575 included a phase-III multi-center, double-blind, parallel-group randomized controlled equivalence trial (INJ-9) of 479 CKD5 patients being treated with reference epoetin-α (Eprex^®^/Erypo^®^, Janssen Cilag/Ortho Biotech Horsham, PA, USA) and randomized to either be converted to HX575 or continue with the reference medicine [5, 6, 7, 11]. This trial showed that ESA-treated patients randomized to being converted to treatment with HX575 or continuing on the reference medication were dosed similarly and achieved similar Hb outcomes. This trial was followed by an EMA-mandated safety observational post-approval study (Epo-PASS) that enrolled 1,695 CKD patients, with or without dialysis, treated with HX575 for 6 months [12]. These studies demonstrated therapeutic equivalence and comparable safety profile to the reference medicine [11] as well as comparable effectiveness and safety to a wide variety of ESAs [12]. 

The MONITOR-CKD5 study [13] was a prospective observational study of hemodialysis patients treated with intravenous (IV) biosimilar HX575. The study aimed to evaluate treatment patterns with biosimilar HX575, associated effectiveness and safety outcomes, and determinants thereof using a multilevel modelling approach [14]. With patients followed for up to 24 months, MONITOR-CKD5 is the largest and longest study of a biosimilar epoetin-α in the hemodialysis setting. 

## Materials and methods 

The methodology of the MONITOR-CKD5 study has been described elsewhere [13]. Key elements are summarized below. The online Supplemental Materials section provides data on the participating centers, definitions of the safety and evaluable samples, data collected at baseline and subsequent visit, safety assessment, statistical analysis, and ethics. As this was an observational, noninterventional study, there were no mandatory assessments or laboratory tests. All data were available from daily clinical practice. 

MONITOR-CKD5 was designed as an international, prospective, longitudinal, observational, pharmacoepidemiological study of hemodialysis patients whose treating physicians prescribed IV HX575 per their best clinical judgment, but informed by local and regional clinical practice guidelines. Patients were recruited from 114 centers in 10 European countries as summarized in Supplemental Table S1. Eligible were male or female CKD5 adult (age ≥ 18) patients on hemodialysis with either original or grafted kidneys, diagnosed with renal anemia, and treated with commercially-available IV HX575. The safety sample consisted of all patients who received at least 1 dose of HX575. The evaluable sample was limited to patients for whom baseline and at least 1 follow-up visit with a valid Hb measurement were available, and the sample also excluded patients with major study protocol violations. 

The primary effectiveness outcome was Hb (g/dL) at baseline and follow-up visits. We also calculated at each follow-up visit the Hb change from baseline, as well as the erythropoietin resistance index (ERI), defined as the ratio of HX575 dose (IU/kg/week) to Hb (g/dL). Patients with ERI > 15 were considered hypo-responsive. They were further classified as chronic hypo-responsive if they had ERI > 15 for 4 or more consecutive months, or nonchronic hypo-responsive if there was no series of 4 consecutive months with ERI > 15. 

Two sources of safety data were used (see also Supplemental Material). First, site investigators recorded at visits 2 – 24 the occurrence of prespecified clinical events since the prior visit. Second, as MONITOR-CKD5 was a study in routine clinical practice, site investigators were instructed to report adverse events (AEs) using standard spontaneous reporting forms for all patients who received at least 1 dose of HX575. 

## Results 

### Patients 

**Figure 1.**
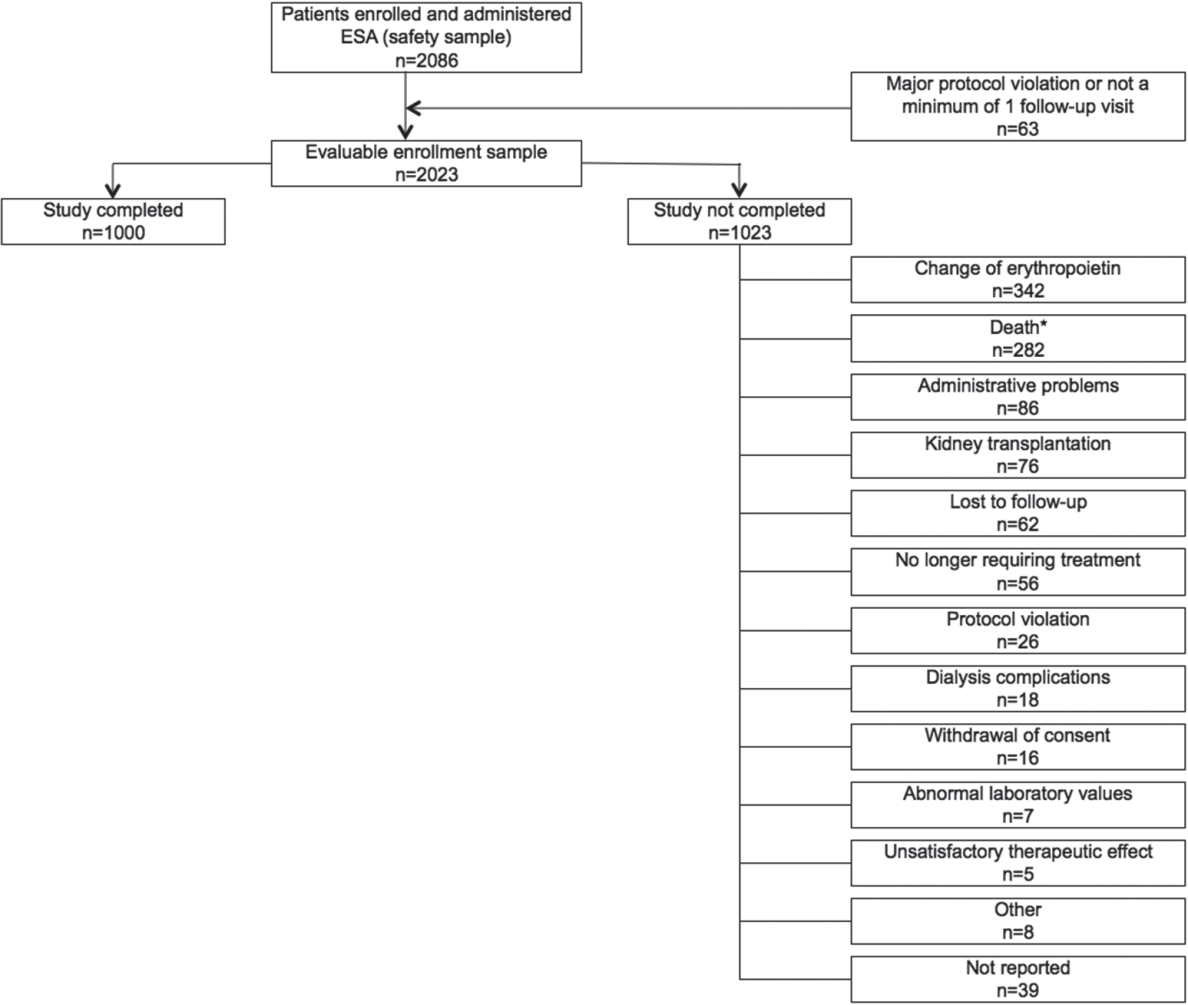
Patient disposition. *Total number of deaths = 288; death as reason for premature study stop = 282; death occurred at study end in 6 patients. ESA = erythropoiesis-stimulating agent.

A total of 2,086 patients were enrolled and received at least 1 dose of HX575; this constituted the safety sample. Of these, 63 were removed because of either major protocol violations (e.g., violation of any inclusion or exclusion criteria, failure to be treated with HX575 at baseline visit, concomitant use of another ESA) or not having a minimum of 1 follow-up visit (Figure 1). This yielded an evaluable enrollment sample of 2,023 patients. Of these, 1,000 patients (49.4%) completed all follow-up visits, while the remaining 1,023 patients (50.6%) ended the study prematurely. Together, change in erythropoietic agent, no longer requiring ESA treatment, kidney transplantation, or death accounted for 73.9% of the reasons for premature study stop. 

In the evaluable sample, most patients (40.6%) were treated in Germany, followed by Romania (18.6%) and Italy (15.3%), accounting together for 74.5% of the study population (Supplemental Table S2). In terms of region, 74.7% of patients came from Western European and the remaining 25.3% from Central and Eastern European countries. The sample was predominantly male (59.3%). Median age was 68 years (range 20 – 93), and the sample was almost exclusively Caucasian (96.2% of 1,960 patients, excluding 63 patients from France, where per national regulations race/ethnicity cannot be collected). The most prevalent primary CKD etiologies included diabetic nephropathy (25.4%), chronic glomerulonephritis (20.4%), and renal vascular disease (16.4%). Median time on dialysis was 2.1 years (range 0 – 35). Most patients (83.4%) had vascular access through a fistula. Relevant major medical history and/or comorbidities included hypertension (81.0%), coronary disease (35.1%), type II diabetes mellitus (30.2%), peripheral vascular disease (19.8%), heart failure (17.1%) and, of note, neoplasia (7.3%). Of the 1,008 patients for whom data were available, 27.0% were iron deficient. The most common concomitant medications were antihypertensive (65.3%), phosphate-binding (61.1%), and antiplatelet (42.2%) agents. At enrollment, most patients (82.5%) had been treated with an ESA (including 56.8% with HX575) and therefore can be considered in the maintenance phase of renal anemia management; whereas the remaining 17.5% were ESA-naïve at baseline, and going through an anemia-correction phase first before transitioning to maintenance therapy. Mean (± standard deviation (SD)) Hb at enrollment was 11.09 (± 1.14), and 68.0% of patients had Hb values in the 10 – 12 g/dL target range. 

### Treatment patterns 

For most patients (75.5%), the lower limit of the individualized target Hb range prespecified by their treating physician was ≥ 10 g/dL but < 11 g/dL; whereas for 5.3% patients it was Hb < 10 g/dL, and Hb ≥ 11 g/dL for the remaining 19.2%. Also, for 74.4% of patients the prespecified individualized upper limit of the target, Hb range was ≥ 11.5 g/dL but < 12.5 g/dL; with 6.5% below and 19.1% exceeding that range. However, only 61.2% of patients met their individualized target range at visit 2 and only 59.4% at visit 24. 

Duration of treatment with HX575 was calculated as the difference in days from the last to the first available patient visit plus 1 day. Median HX575 treatment duration was 505 days or 1.4 years. 

**Figure 2.**
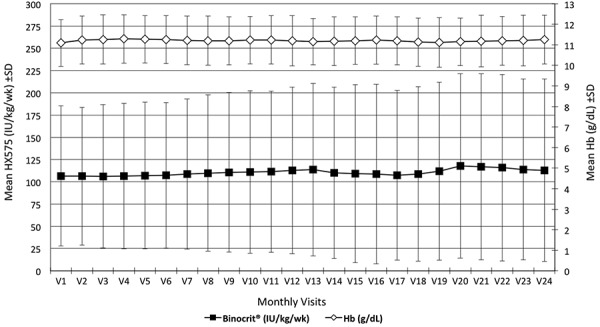
Binocrit^®^ dose and Hb evolution. IU/kg/wk = international units per kilogram per week; SD = standard deviation; Hb = hemoglobin; g/dL = grams per deciliter.

Mean (± SD) HX575 dose rose nominally from 106.5 (± 78.7) international units (IU)/kg/week at enrollment to 113.0 (± 102.5) IU/kg/week at month 24, with a low of 106.1 (± 80.6) IU/kg/week at month 3 and a high of 117.7 (± 103.5) at month 20 (Table 1) (Figure 2). Median dose was 86.7 IU/kg/week at baseline and 85.6 IU/kg/week at month 24, with a low of 78.9 IU/kg/week at month 18 and a high of 88.9 IU/kg/week at months 9 and 12. These variations in mean and median dose over time were not statistically significant, indicating stable dosing patterns over the course of the study (both p = not significant (n.s.)). 

Comparing baseline to visit 24, the proportion of patients receiving iron supplementation decreased by –5.5% (p = 0.017). There were statistically-significant increases in the proportions of patients prescribed vitamin B12 (+5.2%; p = 0.004), vitamin C (+4.9%; p = 0.012), vitamin D (+8.3%; p = 0.001), and folate (+5.3%; p = 0.037), but not vitamin E (Supplemental Table S3). 

At enrollment, 2.8% of patients had previously received a blood transfusion for renal anemia. Over the ensuing 23 months, transfusion rates varied between a low of 0.3% (visit 22) and a high of 1.2% (visit 3). 

### Effectiveness 

Mean (± SD) Hb levels increased nominally from 11.09 (± 1.41) IU/kg/week at enrollment (also the low in the 24-month series) to 11.25 (± 1.19) g/dL at month 24, with a high of 11.29 (± 1.18) at month 4 (Table 2) (Figure 2). Median Hb was 11.1 g/dL at baseline and 11.3 g/dL at month 24, with all median values ranging from 11.1 to 11.3. The absolute values for the mean change in Hb values relative to baseline at months 2 – 24 varied from 0.06 (visits 18 and 20) to 0.18 (visit 4). The corresponding absolute values for median change varied from 0.0 (visits 8, 9, 13, and 14) to 0.2 (visit 24) (Supplemental Table S4). These variations over time were not statistically significant (all p = n.s.); indicating stable Hb levels over time relative to baseline. 

Mean (± SD) ERI scores rose nominally from 9.95 (± 7.78) at baseline to 10.55 (± 10.22) at visit 24, and median ERI declined nominally from 7.82 to 7.76, but these changes were not statistically significant (both p = n.s.) (Supplemental Table S4). Proportions of patients classified as hypo-responsive were 19.5% at baseline and 20.3% at 24 months (p = n.s.). Using ERI > 15 for ≥ 4 consecutive months as criterion, 133 patients (8.0%) were considered chronically hypo-responsive at some time during the study. 

At baseline, out of 1,008 patients for whom data were available, 73.0% had adequate iron stores, but 22.2% had functional and 4.8% had absolute iron deficiency. At month 24, for 131 patients with data available, these proportions were 69.5%, 26.0%, and 4.6%, respectively. These shifts in proportions from baseline to visit 24 were not statistically significant. 

### Safety 

Data were collected at every visit related to thromboembolic events (TEEs), non-TEE events, hospitalizations, and deaths that occurred since the previous visit. In the evaluable sample (n = 2,023), 291 patients (14.4%) experienced a total of 319 TEEs (Supplemental Table S5). AEs were spontaneously reported on all patients receiving at least 1 dose of HX575 (safety sample n = 2,086). Per these reports, 140 patients (6.7%) experienced 254 AEs, including 19 events in 16 patients (0.8%) stated to be related to HX575 treatment (Supplemental Table S6). Of all AEs, 148 occurring in 108 patients (5.2%) were reported as serious (SAE), including 12 events in 11 patients (0.5%) stated to be HX575-related. No patients were reported to have developed non-neutralizing or neutralizing anti-epoetin antibodies or pure red cell aplasia. 

## Discussion 

The 24-month MONITOR-CKD5 study demonstrates that the efficacy of IV HX575, a biosimilar epoetin-α, in managing renal anemia in hemodialysis patients, established in the pivotal randomized controlled trial INJ-9 [5, 11] and validated in the follow-on Epo-PASS commitment study [12], translates into real-world effectiveness with safety evidence extending up to 2 years. Unique to our study, compared to INJ-9 and Epo-PASS, we calculated the ERI to find that the proportion of hypo-responsive patients was about constant at 20% from study start to end. Although INJ-9, Epo-PASS, and MONITOR-CKD5 differed in their reporting of HX575 dosing, dosing was rather consistent across studies. In summary, Hb concentrations and treatment-response indicators converged across these 3 HX575 studies and this under similar dosing regimens. 

Importantly, what our study adds is that this pattern of stable dosing and stable Hb effectiveness outcomes can be achieved sustainably for up to 24 months with HX575 under conditions of greater heterogeneity in patients, clinicians, and settings compared to the relative homogeneity that characterizes efficacy trials – and this in Western as well as in Central and Eastern European countries. Our effectiveness outcomes are also consistent with those of the hemodialysis patients in two international prospective observational studies of anemia-treatment patterns and outcomes with reference epoetin-α: the 5 Western European countries initially involved in the Dialysis Outcomes and Practice Patterns Study (EuroDOPPS), with 4,591 hemodialysis patients from 101 dialysis centers in 5 countries [15], and the European Survey on Anaemia Management (ESAM) with initially 13,121 hemodialysis patients from 792 centers in 14 Western European countries [16, 17, 18]. 

Recombinant human erythropoietin was approved for reducing the need for RBC transfusions in anemic CKD patients. In the pivotal INJ-9 trial, on-study transfusion rates were 1.0% in the HX575-treated group in the 28-week randomized study; rising to 7.4% when including the 56-week open-extension study [5, 11]. In the post-approval Epo-PASS study, monthly transfusion rates varied from 0.3 to 0.7%. It is unclear whether in these 2 studies transfusions were for renal anemia, for other causes, or were “all-cause” transfusions. In our study, monthly post-enrollment transfusion rates specifically for renal anemia were between 0.3% (visit 22) and 1.2% (visit 3). In addition, 107 patients (5.3%) received RBC transfusions on-study for reasons other than renal anemia. Thus, our study provides the first evidence of sustained minimal transfusion rates required to correct renal anemia for up to 24 months of treatment with HX575. 

The proportions of patients with adequate iron stores versus those with functional and absolute iron deficiency remained statistically similar over the 24-month period. There was, however, a significant decline from 66.5 to 61.0% in the proportion of patients receiving iron supplementation. The pivotal trial and Epo-PASS reports do not provide data on iron supplementation in conjunction with HX575 treatment [5, 11, 12]. Likewise, these reports [5, 11, 12] do not relate vitamin supplementation rates. In our study, supplementation with hydrosoluble vitamins was in accordance with current recommendations. Future studies may be necessary to better understand when and how HX575, iron administration, and vitamin supplementation should be aligned. 

While best practice guidelines advise to individualize ESA treatment and set a specific Hb target range for each patient, only ~ 60% of patients were within their target range over the course of MONITOR-CKD5. In fact, as Gesualdo et al. [19] reported, in the post-TREAT era, investigators in the MONITOR-CKD5 study claimed to have a theoretical target Hb range for patients with (lower target) and without (higher target) such risk factors as hypertension, cardiovascular disease, history of stroke, diabetes, prior cancer, or being elderly. However, actual Hb targets achieved across visits tended to fall between 10.0 and 11.9 g/dL, consistently and rather indiscriminately, therefore, suggesting a universal rather than an individualized approach to setting Hb targets. 

On the safety side, 8.1% of patients experienced a shunt thrombosis, 17.1% were hospitalized, and 16.9% died over the 24-month period. No cases of anti-epoetin antibodies or pure red cell aplasia were reported, although one study reported a rate of 2% of patients developing non-neutralizing anti-epoetin antibodies [20]. In addition, there is no evidence that non-neutralizing antibodies play a significant clinical role; neither are there firmly-established reference levels. More generally, the safety signals in MONITOR-CKD5 are in line with those observed in the pivotal trial and Epo-PASS [5, 11, 12] but also those in the EuroDOPPS [15] and ESAM [16, 21] studies. The safety profile of HX575 should be highlighted, especially considering the variation observed in some epoetins claimed to be biosimilar [22]. 

With the relative effectiveness and safety of HX575 established, policy, financing, and access to care issues can be addressed. In an era of payment constraints to dialysis providers, the use of biosimilar epoetin-α can generate significant savings. In the US, the introduction of bundled payments has led dialysis providers to revert back, at least in part, to red blood cell transfusions to manage renal anemia [23]. While this may afford very short-term savings, economic analyses are necessary that compare not only the cost of care but also incorporate outcomes achieved, AEs associated with transfusions, and the opportunity cost of transfused patients not matching for transplantation due to allosensitization [24]. More generalizable across countries and healthcare systems is the approach, as demonstrated for growth factors in chemotherapy-induced anemia and neutropenia, of replacing the use in supportive cancer care of reference medicines with cost-efficient biosimilars [25, 26] and applying the savings generated from such conversion to providing greater patient access to primary anticancer therapy on a budget-neutral basis [27, 28]. 

The MONITOR-CKD5 study has limitations. It was not a population-based study nor did it randomly select sites and, within sites, patients. Selection bias cannot be excluded. Germany, Italy, and Romania provided most patients, and this may have exerted extra weight in the analyses. All patients were treated with HX575, and there was no comparison group of patients treated with other ESAs (not being treated at all with ESAs would have been ethically unacceptable). While limited to centers using HX575, efforts were made to enroll a variety of centers from each country with regards to patient volume, patient mix, and affiliation. In keeping with prevailing guidelines, setting target hemoglobin concentrations as well as dose and frequency of administration of HX575 was left to the discretion of the treating physician. Some rates reported here are visit-specific and influenced by the patients in the study at a given visit, a denominator that declined over time. 

## Conclusion 

The MONITOR-CKD5 study of hemodialysis patients underscores the real-world effectiveness of HX575, a biosimilar epoetin-α, in managing renal anemia. Patients treated for up to 24 months with HX575 showed Hb outcomes equivalent to reference epoetin-α under dosing patterns similar to the reference medicine. The majority of treated patients were maintained within guideline-recommended target Hb ranges. No unknown safety signals, including immunogenicity, were detected. 

## Acknowledgments 

The authors acknowledge the contributions of L. Gesualdo, co-chair of the study steering committee, who resigned after having been elected president of the Società Italiana di Nefrologia. 

## Funding 

The MONITOR-CKD5 study was funded by Hexal AG. The sponsor participated in the development of the protocol, the discussion of the results, and review of the manuscript for scientific content. 

## Conflict of interest 

GL, JM, DG, CC, FD, and FZ received compensation from Hexal AG for their participation in the work reported here. AK and NH are employees of Hexal AG. KM and IA are affiliated with Matrix45. By company policy, they cannot hold equity in sponsor organizations and cannot receive direct personal benefits, financial or other, from sponsor organizations. Matrix45 provides similar services to other biopharmaceutical companies without exclusivity constraints. 

**Table 1.** Treatment patterns.

Binocrit^®^ dose (IU/kg/week)			
Month	n*	M ± SD**	Mdn***
1	1,734	106.5 ± 78.7	86.7
2	1,623	106.3 ± 77.4	85.7
3	1,536	106.1 ± 80.6	85.2
4	1,445	106.4 ± 81.7	84.4
5	1,350	107.1 ± 82.5	83.9
6	1,335	107.3 ± 81.9	84.5
7	1,294	108.7 ± 84.5	86.2
8	1,223	109.8 ± 87.8	87.4
9	1,184	110.6 ± 89.7	88.9
10	1,131	111.1 ± 91.4	87.7
11	1,090	111.4 ± 90.6	88.3
12	1,044	112.7 ± 93.6	88.9
13	938	113.7 ± 96.9	84.8
14	890	110.0 ± 96.3	82.9
15	840	109.0 ± 99.9	82.0
16	796	108.7 ± 101.0	81.4
17	752	107.5 ± 95.4	82.1
18	725	108.9 ± 98.1	78.9
19	649	112.0 ± 100.0	82.8
20	584	117.7 ± 103.5	87.2
21	544	116.8 ± 104.5	86.3
22	519	115.9 ± 104.8	85.1
23	507	113.8 ± 101.5	85.6
24	481	113.0 ± 102.5	85.6

*Evaluable reported weekly doses; **p = n.s. for test of means; ***p = n.s. for test of medians. IU/kg/week = international units per kilogram per week; M = mean; SD = standard deviation; Mdn = median; n.s. = not significant.

**Table 2.** Evolution of treatment outcomes.

Hemoglobin levels			
Month	n*	M ± SD**	Mdn***
1	2,022	11.09 ± 1.14	11.1
2	1,956	11.24 ± 1.17	11.3
3	1,888	11.26 ± 1.19	11.3
4	1,817	11.29 ± 1.18	11.3
5	1,736	11.27 ± 1.15	11.3
6	1,709	11.25 ± 1.17	11.3
7	1,691	11.22 ± 1.18	11.2
8	1,620	11.20 ± 1.19	11.2
9	1,608	11.20 ± 1.17	11.2
10	1,546	11.23 ± 1.16	11.3
11	1,502	11.24 ± 1.18	11.2
12	1,446	11.20 ± 1.22	11.3
13	1,314	11.16 ± 1.13	11.2
14	1,237	11.17 ± 1.19	11.2
15	1,211	11.20 ± 1.15	11.3
16	1,126	11.23 ± 1.17	11.2
17	1,088	11.19 ± 1.17	11.2
18	1,035	11.13 ± 1.18	11.2
19	968	11.12 ± 1.21	11.1
20	879	11.15 ± 1.16	11.2
21	836	11.18 ± 1.26	11.2
22	803	11.19 ± 1.19	11.2
23	780	11.21 ± 1.23	11.3
24	759	11.25 ± 1.19	11.3

*Evaluable observations; **p = n.s. for test of means; ***p = n.s. for test of medians. M = mean; SD = standard deviation; Mdn = median; n.s. = not significant.
